# Infants’ representations of the infant body in the first year of life: a preferential looking time study

**DOI:** 10.1038/s41598-023-41235-w

**Published:** 2023-08-28

**Authors:** Silvia Rigato, Maria Laura Filippetti, Carina de Klerk

**Affiliations:** Centre for Brain Science, Department of Psychology, University of Essex, Wivenhoe Park, Colchester, CO4 3SQ UK

**Keywords:** Human behaviour, Sensory processing

## Abstract

Representing others’ bodies is of fundamental importance for interacting with our environment, yet little is known about how body representations develop. Previous research suggests that infants have expectations about the typical structure of human bodies from relatively early in life, but that these expectations are dependent on how closely the stimuli resemble the bodies infants are exposed to in daily life. Yet, all previous studies used images of adult human bodies, and therefore it is unknown whether infants’ representations of *infant bodies* follow a similar developmental trajectory. In this study we investigated whether infants have expectations about the relative size of infant body parts in a preferential looking study using typical and disproportional infant bodies. We recorded the looking behaviour of three groups of infants between 5 and 14 months of age while they watched images of upright and inverted infant bodies, typical and proportionally distorted, and also collected data on participants’ locomotor abilities. Our results showed that infants of all ages looked equally at the typical and proportionally distorted infant body stimuli in both the upright and inverted conditions, and that their looking behaviour was unrelated to their locomotor skills. These findings suggest that infants may need additional visual experience with infant bodies to develop expectations about their typical proportions.

## Introduction

From birth, our bodies provide the main tool for interacting with the external environment, and thus the development of infants’ bodily abilities is fundamentally linked with their ability to interact with, and learn from, the world around them^1^. How infants represent this ever-present part of their existence is a fascinating question that has remained largely unanswered. Previous research on body representation development has focused on infants’ expectations and understanding about typical body configurations, though largely using adult bodies as the stimuli. These studies have demonstrated that infants differentiate between pictures of typical and scrambled adult body configurations from about 15 months of age^2^, and that around 14 months of age infants begin to show a differential neural signature of body processing when they observe images of upright versus inverted bodies^3^. Furthermore, a study by Heron and Slaughter^4^ reported that when real-live human models or mannequins, instead of pictures, were used even 9-month-old infants could differentiate between typical and scrambled adult body configurations. However, the literature is still controversial in that there is also evidence that suggests that infants are sensitive to the overall organisation of body parts from as early as 3 months of age^5^. In this study, Gliga and Dehaene-Lambertz^5^ directly compared intact and scrambled face and body stimuli and found that one of the face- and body-sensitive ERP components—the P400—was reduced by stimuli whose first-order structure had been changed relative to intact stimuli, therefore suggesting structural encoding of the face and human body form from 3 months of age. The discrepancies in the results of these studies may be due to experimental differences. For example, while the looking time studies^2, 4^ used symmetrical distortions of bodies without altering the head position, the ERP study^5^ used an asymmetrical distortion in which a limb was placed in the location of the head. Young infants frequently see upper body parts and spend a lot of time focusing on faces^6^. This experience may help them form expectations about the typical configuration of the head’s position in relation to the torso, resulting in a differential neural response to the normal and distorted body.

The studies reviewed so far focused on infants’ expectations and understanding of the organisation of body parts. However, body proportions are another significant source of information that adults use to distinguish between human bodies (e.g.^7, 8^). A series of studies by Zieber and colleagues, in which the proportions of the body were manipulated by lengthening the neck and torso and shortening the legs, suggests that an understanding of the typical proportions of adult human bodies is present at different ages depending on the experimental paradigm used. Nine-month-olds, but not 5-month-olds, displayed a preference for the typical body in a spontaneous preferential looking task^9^ (although note that infants as young as 3.5-month-old exhibited a preference for the typical body when a habituation task was used^10^). Crucially, infants did not show a preference when the body stimuli were presented in an inverted configuration, leading the authors to conclude that 9 months of experience is enough for infants to develop expertise with bodies to generate a spontaneous orientation-specific preference.

Together, these studies suggest that infants have expectations about the first-order structure of bodies from relatively early on, but that these expectations may depend on how closely the stimuli resemble the bodies infants observe in daily life. Therefore, they seem to indicate a key role of the visual experience with bodies infants accumulate in their first months of life. However, all these previous studies used adult human bodies, and it is unknown whether infants’ representations of infant bodies—bodies that more closely resemble their own—follow a similar developmental trajectory and rely on the same type of experience. Examining this question is an essential first stepping stone to further our understanding of the mechanisms underlying the development of infants’ own body representations. In the current study, we investigated whether infants have expectations about the relative size of infant body parts in a preferential looking study using typical and disproportional infant bodies.

According to the account we put forward in^11^, the visual, motor, and proprioceptive experience that infants obtain while they observe their own full body may play a critical role in the development of infants’ ability to form expectations about their own body’s configuration. There is some preliminary evidence that infants’ ability to represent the various parts of their body indeed depends on the amount of multisensory experience they have acquired with these body parts. For example, a tactile-localization study^12^ in which vibrating stimuli were applied to different points on the head and arms of 7- to 21-month-old infants, showed that infants were able to localize targets on body parts for which they have accumulated more multisensory experience, i.e. near the mouth and on the hand, at a younger age than targets for which they have likely obtained significantly less correlated multisensory experience, i.e. near the ear or on the forehead. One possibility is that infants’ representations of their own full body are similarly influenced by the amount of accumulated full body multisensory experience. Supporting evidence comes from a study by Slaughter et al.^2^ that showed that walking 12-month-olds were able to discriminate typical from scrambled body configurations, while to non-walking 12-month-olds were not (but also see^13^). Infants who locomote have increased opportunities to use their whole body in a coordinated fashion (e.g. crawling, walking) and thus for integrating proprioceptive, tactile, and visual experiences (for similar discussion see^3, 6^). In the present study, we examined whether, as infants learn to crawl and walk, these multisensory experiences influence their ability to discriminate between images of typical and distorted infant bodies.

The current study aimed to replicate and extend the previous work by Zieber and colleagues^9^ using pictures of infant bodies instead of adult bodies. We used a highly similar design to^9^, and observed 5-, 9- and 12- to 14-month-old infants’ spontaneous looking preference for typically configured and proportionally distorted infant bodies. We reasoned that if the 9-month-old infants’ preference for the typically proportioned adult bodies in the Zieber et al.^9^ study was based on their previous visual experience with seeing typical adult bodies, then we should not find a preference for the typical over the distorted infant bodies in the pre-walking infants—as these infants are unlikely to have obtained significant visual experience with infant bodies from an angle that would allow them to learn about their relative proportions. Walking infants, on the other hand, are more likely to obtain visual experience with typical infant body proportions by observing their own reflection. However, if multisensory experience, rather than just visual experience, with one’s own body aids the development of expectations about typical body proportions, then we would expect to find a preference for the upright typical over the disproportional infant body in those infants who have accumulated more experience with using their whole body, for example by crawling or walking, i.e. in 9- and 12- to 14-month-olds, but not in the younger infants. The predictions for the 5- and 9-month-old infants were preregistered (https://aspredicted.org/MZT_1BH).

## Methods

### Participants

A total of 104 parents accessed the study at home via Lookit (Children Helping Science, https://childrenhelpingscience.com/). Of these, 9 infants did not complete the study and 16 were outside the age ranges used in the study and could therefore not be included in the analyses. Two additional 9-month-olds and four 12- to 14-month-old infants were excluded from the analyses (one because did not provide valid data in at least one upright and one inverted trial, 3 because the parent or a sibling interfered, one for developmental delays, and one for complications at birth). The sample reported on this paper therefore consisted of 25 5-month-old infants (11 females, age range 136–179 days, M = 158.8 days; SD = 10.9 days); 23 9-month-old infants (14 females, age range 245–288 days, M = 274 days; SD = 11.9 days), and 25 12- to 14-month-olds (13 females, age range 345–440 days, M = 396.5 days; SD = 22.7). The required sample size of 23 participants per age group was determined through a power analysis in GPower using the smallest effect size of interest and at least 80% power to detect a significant effect with an alpha level of 0.05 (we used partial eta squared 0.06: a medium effect—note that Zieber et al.^9^ found a partial eta squared of 0.09 for the interaction between age and condition).

Participants were recruited through Lookit as well as through the University of Essex Babylab database. Ethical approval was gained from the University of Essex Ethics Sub-Committee (ID ETH 1920-1221). All methods were performed in accordance with the Declaration of Helsinki. All participating infants were born full-term (at 37 weeks of gestation or later). Infants’ ethnic backgrounds were: White (60.3%), White mixed with another ethnic background (30.1%), Hispanic (4.1%), Asian (2.8%), Black or African American (1.4%) and one did not specify (1.4%). Fourteen mothers reported their age to be in the range of 25–29 years, 23 in the range 30–34 years, 29 in the range 35–39 years, and 7 in the 40–44 age range. Family income ranged between $20,000 and over $200,000 (average $109,536).

### Stimuli

**Figure 1:**
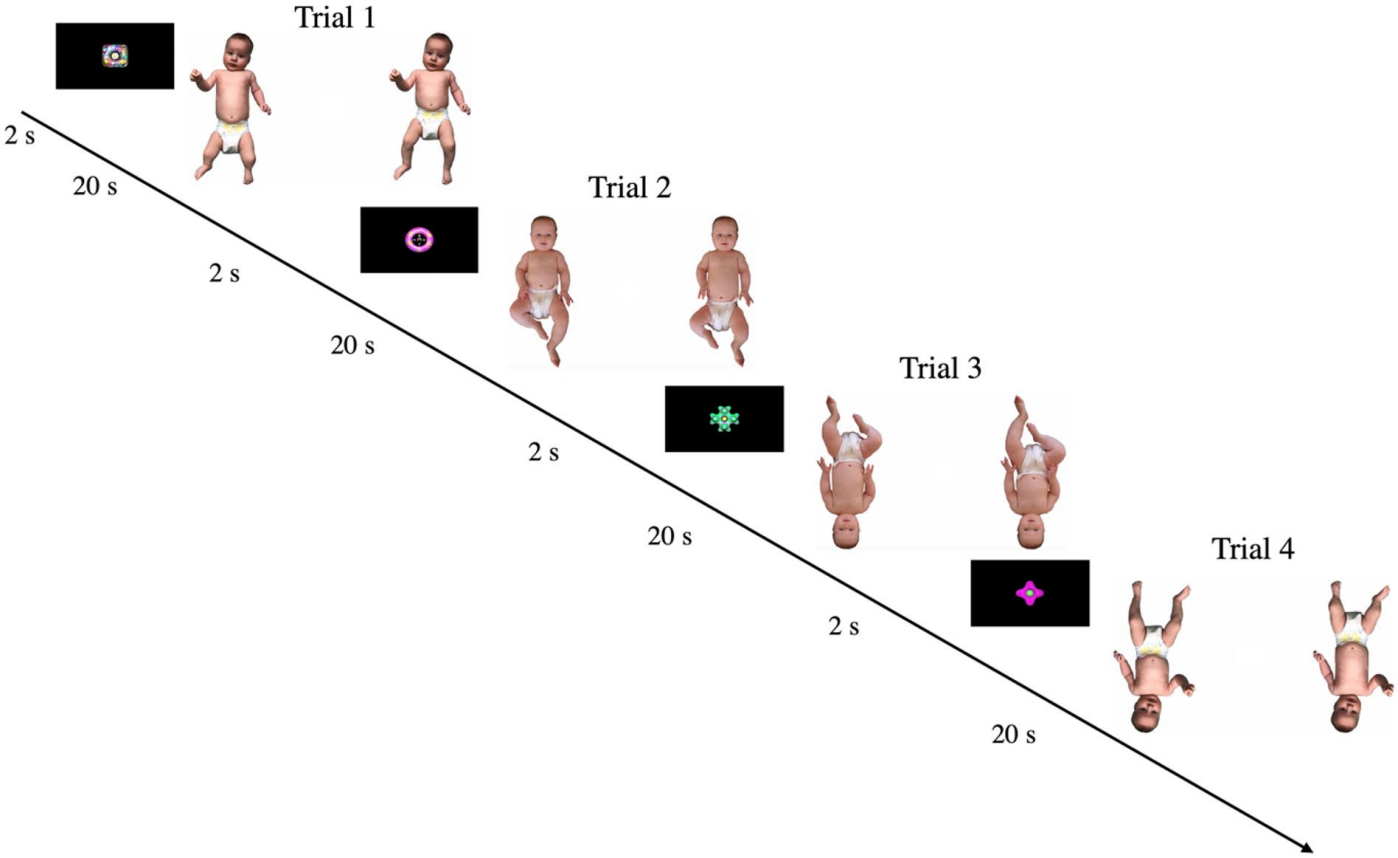
Looking time paradigm used in the study. Note: A central attention getter (2 s) was followed by a pair of infant body images (20 s), one typical and one proportionally distorted, either upright or inverted.

In the current study, we used a within-subjects design in which all infants were shown images of typical and proportionally distorted infant bodies presented upright and inverted (see Fig. 1). The distorted bodies were created from images of two typical infant bodies, a male 4-month-old and a female 5-month-old, wearing only a nappy, by elongating the torso and shortening the legs, while maintaining the same body height as the normal body. Note that while in the Zieber et al.^9^ study the model’s neck was elongated as well, the infant stimuli did not have a discernable neck to stretch. The proportional distortion of the bodies (leg length decreased by approximately 8.33% and torso length increased by approximately 8.33%) was the same as the stimuli in Zieber et al.^9^.

The experiment included two trials for each of the conditions (upright and inverted for each of the model babies). The infants always saw two upright and two inverted trials in a row but the order of these two conditions were counterbalanced across infants. The initial left–right position of the normal and distorted bodies was counterbalanced across infants. In addition, the left–right position of the normal and distorted bodies was switched across the test trials to avoid a side bias. In total, four counterbalancing orders were created.

### Visual preference task

Looking data were collected via webcam using the Lookit platform to assess the infants’ visual preference for the typical infant body in the upright and inverted condition (see Fig. 1). To keep the infant engaged throughout the task, each trial began with a central attention getter, which was presented for 2 s. The fixation stimulus was then followed by the pair of body images, one typical and one distorted, either upright or inverted, which remained on the screen for 20 s. A calm soundtrack was played throughout the experiment. At the beginning of the study, parents were asked to set up their webcam and microphone and to check that their baby’s face was clearly visible. They were then asked to review the consent information and record a short video of themselves giving consent to participate in the study. After this, they had a chance to preview the stimuli making sure their infant was not watching. Finally, parents were asked to sit their baby on their lap facing the screen, to not direct their baby's attention and to close their eyes during the experiment to avoid influencing their baby’s responses. At any point throughout the experiment parents had the option to pause the experiment by pressing the space bar in case the infant looked away from the display or became fussy or inattentive, and to resume the experiment when the infant was happy to continue to watch at the screen.

### Motor abilities questionnaire

At the end of the stimuli presentation, parents were asked to complete a short questionnaire on their infant’s motor skills. They were asked to report whether their infant was able to stand, cruise, crawl, and walk, and if yes, for how long (in number of weeks). For the purpose of this study, we focused on the ability to crawl in the 9-month-olds group and the ability to walk in the 12- to 14-month-olds group. Sixteen of the 9-month-olds were crawlers and 7 were non-crawlers; while 16 of the 12- to 14-month-olds were walkers and 9 were not.

### Data coding and analyses

Looking time data were coded using Datavyu by three independent coders who were unaware of the hypothesis of the study. All coders coded at least 20% of the videos in the 5 and the 9 months age group to determine intercoder reliability. All coders achieved acceptable reliability^14, 15^, all Cohen’s Kappa coefficients κ between 0.57 and 0.87. Each coder was then assigned to code the rest of the participants in one of the age groups.

For statistical analyses, trials were excluded if: (1) the infant did not look for a minimum of 300 ms at each of the two images on the screen; (2) the parent or a sibling interfered (e.g. by pointing to one of the stimuli). We identified several looking times that were shorter than Q1 − 1.5 × IQR or greater than Q3 + 1.5 × IQR (5 months inverted condition: N = 1; 9 months upright condition: N = 1; 9 months inverted condition: N = 1; 12 months upright condition: N = 2; 12 months inverted condition: N = 2). We did not exclude these outliers (as per our preregistration) because natural variation in looking time is expected in infant populations. We calculated the percent preference for the typical body in the upright and inverted condition by dividing the total duration of looking at the typical body across the two trials for each condition (upright and inverted) by the total duration of looking at both the typical and disproportional images across the two trials, and multiplying this ratio by 100. We first performed a repeated measures ANOVA with condition (percent preference for the typical body in the upright vs. inverted condition) as within-subjects factor and age (5 months vs. 9 months vs. 12- to 14 months) and order (four counterbalancing orders) as the between-subjects factors. This last factor was included in the analyses to check for the effect of using a within-subjects design instead of a between-subjects design like in Zieber et al.^9^. We reasoned that, for example, for infants who saw the upright condition first, and showed a preference for the proportional body, this preference may carry over to the inverted condition. In case of a significant order effect, we planned to analyse the data as if the study was run as a between-subjects design, i.e. using only the first two trials presented to each infant (see our preregistration). We also looked for effects of locomotor skills in the two older groups by running an ANOVA for the 9-months group with condition (percent preference for the typical body in the upright vs. inverted condition) as within-subjects factor and ability to crawl as the between-subjects factor, and a separate ANOVA for the 12- to 14-months group with condition (percent preference for the typical body in the upright vs. inverted condition) as within-subjects factor and ability to walk as the between-subjects factor.

## Results

**Figure 2:**
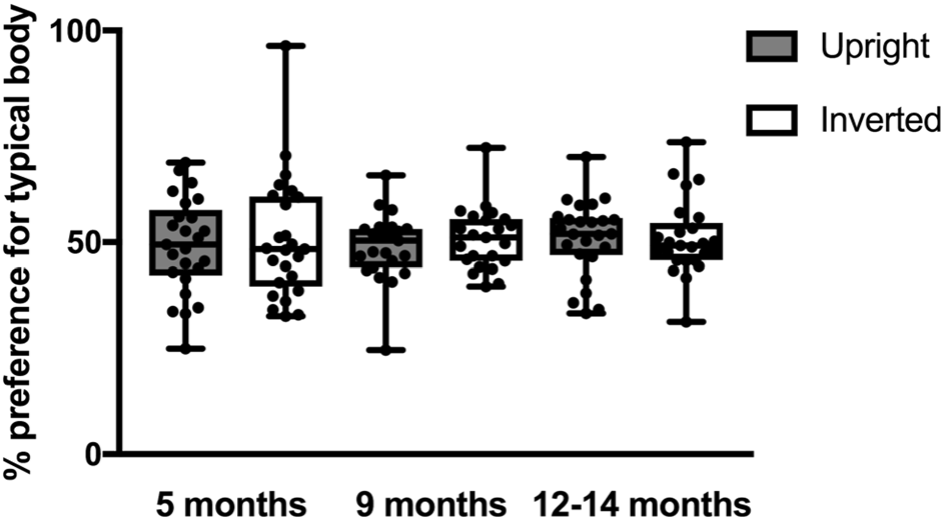
Percent preference for the typically configured infant body in the upright and inverted condition for each age group.

As can be seen in Fig. 2, infants of all ages looked equally at the typical and proportionally distorted body stimuli in both the upright and inverted condition. Indeed, the analyses revealed no effect of condition, *F*(1,61) = 0.342, *p* = 0.561, no interaction with age, *F*(2,61) = 0.113, *p* = 0.893, or with counterbalancing order,* F*(3,61) = 1.081, *p* = 0.364, and no three-way interaction, *F*(6,61) = 0.673, *p* = 0.672. (Results are the same when excluding outliers, i.e. no effect of condition, p = 0.604, no interaction with age, p = 0.450, or with counterbalancing order, p = 0.293, and no three-way interaction, p = 0.460.)

Although we did not find a significant order effect, we also performed an exploratory between-subjects analysis (as in^9^, i.e. using only the first two trials presented to each infant; so only the upright or inverted condition was included for each infant depending on which was presented first). These analyses confirmed that infants of all ages looked equally at the typical and proportionally distorted body stimuli in both the upright and inverted condition (there was no effect of condition, *F*(1,67) = 0.045, *p* = 0.833, no effect of age, *F*(2,67) = 0.223, *p* = 0.800, and no interaction, *F*(2,67) = 0.039, *p* = 0.961).

**Figure 3:**
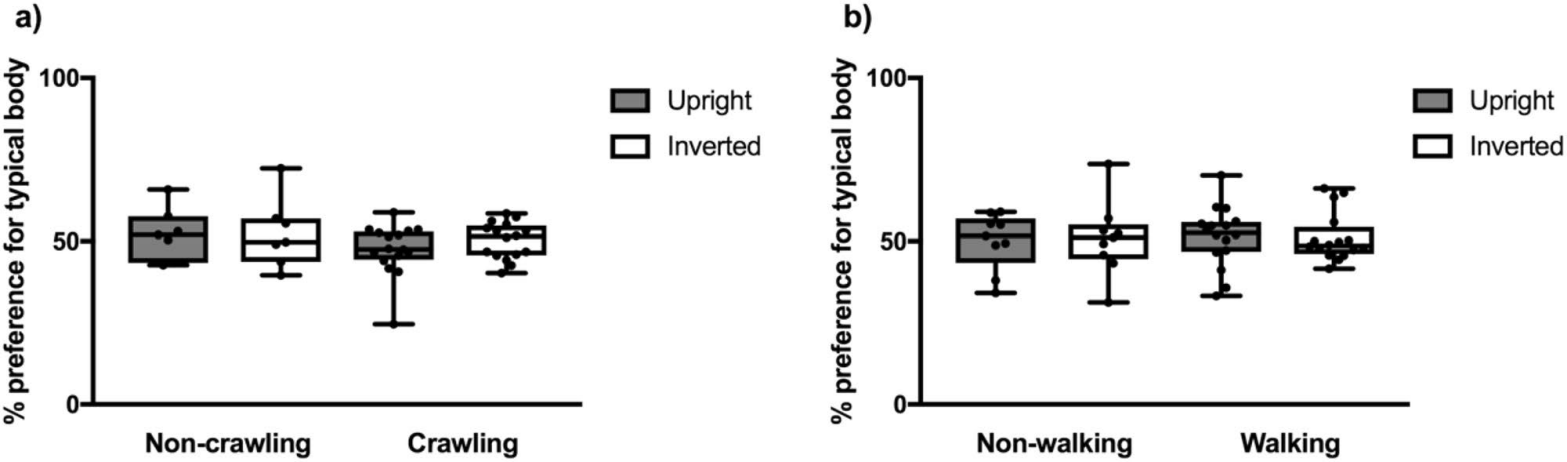
Percent preference for the typically configured infant body in the upright and inverted condition based on locomotor abilities. (**a**) Preference scores for the 9-month-old infants who could and could not crawl at the time of testing and (**b**) preference scores for the 12- to 14-month-old infants who could and could not walk at the time of testing.

We then looked for effects of locomotor abilities. The analyses again revealed no significant main effects or interactions for the 9-month-olds (condition, *F*(1,21) = 0.435, *p* = 0.517, interaction with crawling status, *F*(1,21) = 0.281, *p* = 0.602) or the 12- to 14-month-olds (condition, *F*(1,23) = 0.008, *p* = 0.929, interaction with walking status, *F*(1,23) = 0.043, *p* = 0.838) (see Fig. 3).

## Discussion

With this study, we set out to investigate how infants develop representations of infant bodies. To this aim, we exposed three groups of infants between 5 and 14 months of age to images of upright and inverted typically configured and proportionally distorted infant bodies while recording their spontaneous looking behaviour. We aimed to replicate and extend previous work by Zieber and colleagues^9^ using pictures of infant bodies instead of adult bodies. Our results revealed that infants at all ages looked equally at the typical and distorted body stimuli in both the upright and inverted condition, and that their looking behaviour was unrelated to their locomotor abilities. Therefore, these findings do not support the hypothesis that accumulated multisensory experience with the whole body facilitates a preference for the upright typical body. Instead, it seems that while infants have accumulated sufficient visual experience with adult bodies by 9 months of age^9^, they may need additional visual experience with infant bodies (their own or others) to be able to differentiate between a typically configured and a proportionally distorted infant body. Although infants—especially walking ones—have opportunities to observe their own full body standing in front of a mirror or being around other standing infants, e.g. at a nursery setting, their visual attention may be more focused on the adult caregivers. Even more, since this study was run during the COVID-19 pandemic, opportunities for observing other standing infants may have been limited due to nurseries being closed and/or children having fewer playdates or interactions with peers. Additionally, infant body proportions change significantly over the first few years of life. Therefore, even if infants have sufficient opportunities to obtain visual experience with infant bodies, their proportions are likely highly variable. In relation to this, a limitation of the current study is that, in the effort to keep the stimuli constant across the study, we used stimuli of a 4- and a 5-month-old infant for all age groups. It is possible that we would have obtained different results if we had used stimuli of infants the same age as our older participants.

Another possibility is that infants' difficulty in learning about full-body appearance is the result of an attentional bias in which young infants are first more engaged in the process of building representations of the functions of the human body than in learning and identifying the structural properties of the body^16^. Although we used images of *other* infants’ bodies in the current study, research on the development of *own* body representations indeed suggests that learning about body structure is a lengthy process that is not complete until the third year of life. For example, Brownell, Svetlova and Nichols^17^ suggest that infants first become aware of their own body parts in isolation from 12 months of age, then start to represent their own whole body as an object with a size, shape, and hierarchically organised spatial structure from 18 months, and that finally these various components consolidate and integrate over the preschool years.

The development of other abilities in the second year of life might also contribute to the infants’ ability to represent the structure of infant bodies, and eventually lead to own-body representations. For example, various authors suggest that when infants start to recognise themselves in the mirror, this objectified view of the body, i.e. a third-person representation of the infant's own public body^18, 19^, becomes processed as part of the self^20, 21^. Thus, it could be that in order to use the visual experience they obtain while observing their own bodies in the mirror, infants need to be able to recognise themselves.

Taken together, these lines of research seem to suggest that infants older than 18 months of age—who are starting to have representations of their own whole body and are likely able to recognise themselves in the mirror—should be able to discriminate between images of infant bodies typically configured and proportionally distorted. While this seems plausible, it will not help to disentangle the contribution of visual vs multisensory experience in the development of infant body representations, as by 18 months of age infants would have accumulated a great amount of visual experience with their own as well as other infants’ bodies and at the same time would be expert crawlers and, usually, walkers. Nevertheless, it may be interesting for future research to replicate this work with infants in the second year of life, and map their performance to indices of their developing body-related abilities.

This work is not without limitations. For example, another possible explanation for our findings is that the differences between the stimuli were too subtle for infants to show a preference. However, the modifications applied to the proportions of the body stimuli were the same as those applied to adult body stimuli in Zieber et al.^9^. Yet, it would be interesting for future research to test adult participants familiar and unfamiliar with this kind of stimuli, e.g. parents/carers vs. non-parents/non-carers, in a discrimination task with these same stimuli to understand whether a lack of visual experience can indeed explain our findings. Infants’ limited working memory capacity might have also contributed to the null results. While Zieber et al.^9^ used a between-subject design and only showed infants body stimuli of one individual, in this study we employed a within-subject design and showed infants body stimuli of two identities. Our exploratory analyses showed that the design employed cannot explain our different results, however it is still possible that infants would have expressed a visual preference for one of the stimuli if only one identity was shown across all upright or all inverted trials. It is also important to note that this study was conducted entirely online. While this allowed parents to participate at the most convenient time for their baby, and allowed us to collect data from infants from a large variety of backgrounds while the COVID-19 pandemic prevented in-lab studies, it lacked the control typical of a laboratory setting. Although it would be important for this study to be replicated in the lab to explore whether the same results are obtained in a more controlled setting, recent evidence suggests that the results of online studies are comparable to lab studies^22, 23^. Finally, in this study we used pictures of other infants’ bodies. Specifically, we used two exemplars across all infants, which might limit the generalizability of the findings given that there could be something unique about the proportions or the postures of the selected body images. While this represents a first step towards the understanding of the development of own-body representations, and it was a practical solution for conducting the study entirely online when labs were closed due to the COVID-19 pandemic, a logical next step would be to use images of the infants’ own bodies.

Despite its limitations, this study suggests that infants’ representations of infant bodies, that arguably resemble their own body, follow a different trajectory to the representations of adult bodies^9^, and that this ability does not develop before 14 months of age. Infants may indeed need more visual exposure with their own, or other infants’ bodies to develop the expertise necessary to discriminate between typical and atypical infant body proportions. This ability might also develop in conjunction with, or as a result of, other related skills, such as mirror self-recognition.

## Data Availability

The data are openly available at https://osf.io/nr3dm/?view_only=ce230caf5360427ab1d4258e10a5f3d3. The analyses presented here were preregistered at https://aspredicted.org/MZT_1BH.
